# Supplementary material for: Generation and Selection of a Panel of Pan-Filovirus Single-Chain Antibodies using Cell-Free Ribosome Display
Source: Am J Trop Med Hyg. 2019 May 6;101(1):198–206. doi: 10.4269/ajtmh.18-0658 (PMC6609206; doi:10.4269/ajtmh.18-0658)
Supplement: Supplementary file 1 [file tpmd180658.SD1.pdf]

The following are supplemental materials and will be published online only

```
                                                                CDR1 VH
EbovGPscFv18-1   MAWSHPQFEKTGSGDVMLVESGGGLVQSGGSMELSCVASGITFG-NYWMNRVRQSPEKGLEWVAE
EbovGPscFv35-2   MAWSHPQFEKTGSGDVKLQESGPGLVKPSQSLSLTCSVTGYSITSGYYWNWIRQFPGNKLEWMGY
EbovGPscFv10-3   MAWSHPQFEKTGSGEVQLVESGGGLVQPGGSLSLSCAASGFTFT-DYYMSWVRQPPGKALEWLGF
EbovGPscFv4-2    MAWSHPQFEKTGSG-------GAELVKSGASVKLSCTASGFNIK-DDYMHWVKQRPEQGLEWIGW
EbovGPscFv27-5   MAWSHPQFEKTGSGQVQLQQSGAELVKPGASVKSSCTATGFNIK-DYYIHWVKQRTEQGLEWIGR
EbovGPscFv13-1   MAWSHPQFEKTGSGQVQLQQSGAELVKPGASVKLSCKASGYTFT-EYTIHWVKQRSGQGLEWIGW
EbovGPscFv39-1   MAWSHPQFEKTGSGEVQLQQSGAELMKPGASVKLSCKATSYTFT-GYWIEWVKQRPGRGLEWIGE
EbovGPscFv16-1   MAWSHPQFEKTGSG-------GAELVRSGASVKLSCTASGFNIK-DDYMHWVKQRPEQGLKWIGW
EbovGPscFv3-2    MAWSHPQFEKTGSGQVQLQQPGAELVKPGASVKLSCKASGYTFT-SYWMHWVKQRPGRGLEWIGR
EbovGPscFv22-1   MAWSHPQFEKTGSGQVQLQQPGTELVKPGASVKLSCKASGYTFT-SYWMHWVKQRPGQGLEWIGN
                 **************       *   *:: . *:. :* .:. .:         ::*    . *:*:.

                 CDR2 VH                                            CDR3 VH
EbovGPscFv18-1   IRLKSNNYATHYVESVKGRFTISRDDSKSSVYLRMNNLKAEDTGIYYCTRHYYG--------CMDY
EbovGPscFv35-2   ISYDG---SNNYNPSLKNRISITRDTSKNQFFLKLNSVTTEDTATYYCARESPYYG--SSHWYFGV
EbovGPscFv10-3   IRNKANGYTTEYSASVKGRFTISRDNSQSILYLQMNALRAEDSATYYRARPYYS-----NYVGFAY
EbovGPscFv4-2    IDPENG--DTEYASKFQGKATITADTSSNTAYLQLSSLTSEDTAVYYCTT-YYY-----GSAWFAY
EbovGPscFv27-5   IDPEDG--ETKYAPKFQGKATITADISPNTAYLQPSSLTSEDTAVYYCTRGGYY-----GPWYFDV
EbovGPscFv13-1   FYPGSG--SIKYDEKFKDKATLTADKSSSTVYMELSRLTSEDSAVYFCARHEPYYYGSSYEGPFDY
EbovGPscFv39-1   ILPGSG--STNYNEKFKGKATFTADTSSNTAYMQLSSPTTEDSAIYYCARDY-------YDWYFDV
EbovGPscFv16-1   IDPENG--DTEYASKLQGKATITADTSSNTAYLQLSSLTSEDTAVYYCARPIYYYG--SSHWYFDV
EbovGPscFv3-2    IDPNSG--GTKYNEKFKSKATLTVDKPSSTAYMQLSSLTSEDSAVYYCARTGYY----GSSWYFDV
EbovGPscFv22-1   TNPSNG--GTNYNEKFKSKATLTVDKSSNTAYMQLSSLTSEDSAVYYCARSV--------SGNFAY
                           .*   ..:.:.   *     .    ::. .      :**:.  *: :        :

                             LINKER                            CDR1 VL
EbovGPscFv18-1   WGQGTTVTVSSGGGGSGGGGSGGGGSGGGGSLSLPVSLGDQASISCRSSQNLEDS-NGKTFLNWYL
EbovGPscFv35-2   WGTGTTVTVSSGGGGSGGGGSGGGGSGGGGSASLSASVGETVTITCRTSE------NIYSYLAWYQ
EbovGPscFv10-3   RGQGTLVTVSAGGGGSGGGGSGGGGSGGGGSASLSASVGETVTITCRASE------NIYSYLAWYQ
EbovGPscFv4-2    WGQGTLVTVSAGGGGSGGGGSGGGGSGGGGSASLSVSVGETVTITCRASG------NIHNYLAWYQ
EbovGPscFv27-5   WGTGTTVTVSSGGGGSGGGGSGGGGSGGGGSASLSVSVGETVTITCRASE------NIYSYLAWYQ
EbovGPscFv13-1   WGQGTTLTVSSGGGGSGGGGSGGGGSGGGGSSPLSVSAGEKVTMSCKSSQSLLNSGNQKNYLAWYQ
EbovGPscFv39-1   WGTGTTVTVSSGGGGSGGGGSGGGGSGGGGSSSLSVSAGEKVTMSCKSSQSLLNSGNQKNYLAWYQ
EbovGPscFv16-1   WGTGTTVTVSSGGGGSGGGGSGGGGSGGGGSLSLPVSLGDQASISCRSSQSLVHS-NGNTYLHWYL
EbovGPscFv3-2    WGTGTTVTVSSGGGGSGGGGSGGGGSGGGGSLSLPVSLGDQASISCRSSQSIVHS-NGNTYLEWYL
EbovGPscFv22-1   WGQGTLVTVSAGGGGSGGGGSGGGGSGGGGSLSLPVSLGDQASISCRSSQSLVHS-NGNTYLHWYL
                  *     :***:********************   *  .*  *:  .:::*::*       *   .:* **

                           CDR2 VL                                    CDR3 VL
EbovGPscFv18-1   QRPGQTPQLLIYRVPNRFSGVLDRFSGTGTGTDFTLKISRVEAEDVGVYFCLQVTHVPP--TFGGG
EbovGPscFv35-2   QKQGKSPQLLVYNAKTLAEGVPSRFSGSGSGTQFSLKINSLQPEDFGSYYCQHHYGIPP-WTFGGG
EbovGPscFv10-3   QKQGISPQLLVYNAKTLAEGVPSRFSGSGSGTQFSLKINSLQPEDFGNYYCQHHYTTP--FTFGGG
EbovGPscFv4-2    QKQGKSPQLLVYNAKTLADGVPSRFSGSGSGTQYSLKINNLQPEDFGSYYCQHFWSTP--YTFGGG
EbovGPscFv27-5   QKQGKSPQLLVYNAKTLAEGVPSRFSGSGSGTQFSLKINNLQPEDFGSYYCQRHYDTP--YTFGGG
EbovGPscFv13-1   QKPGQPPKLLIYGASTRESGVPDRFTGSGSGTDFTLTISSVQAEDLAVYYCQNDHSYP--LTFGAG
EbovGPscFv39-1   QKPGQPPKLLIYGASTRESGVPDRLTGSGSGTDFTLTISSVQAEDLAVYYCQNDHSYP--WTFGGG
EbovGPscFv16-1   QKPGQSPKLLIYKVSNRFSGVPDRFSGSGSGTDFTLKISRVEAEDLGVYFCSQSTHVP--WTFGGG
EbovGPscFv3-2    QKPGQSPKLLIYKVSNRFSGVPDRFSGSGSGTDFTLKISRVEAEDLGVYYCFQGSHVPPLYTFGGG
EbovGPscFv22-1   QKPGQSPKLLIYKVSNRFSGVPDRFSGSGSGTDFTLKISRVEAEDLGVYFCSQSTHVP--WTFGGG
                 *:.*   *:**.*  .     .**  .*::*:*:**:::*.*. :: **.. *:* .     *   ***.*

EbovGPscFv18-1   TKLELRRADAAPTVSIFPPSSEQLTSGGASVVCFLNNFTHCQELQQDECS
EbovGPscFv35-2   TKLEIKRADAAPTVSIFPPSSEQLTSGGASVVCFLNNFTHCQELQQDECS
EbovGPscFv10-3   TKLEIKRADAAPTVSIFPPSSEQLTSGGASVVCFLNNFTHCQELQQDECS
EbovGPscFv4-2    TKLEIKRADAAPTVSIFPPSSEQLTSGGASVVCFLNNFTHCQELQQDECS
EbovGPscFv27-5   TKLEIKRADAAPTVSIFPPSSEQLTSGGASVVCFLNNFTHCQELQQDECS
EbovGPscFv13-1   TKLELKRADAAPTVSIFPPSSEQLTSGGASVVCFLNNFTHCQELQQDECS
EbovGPscFv39-1   TKLEIKRADAAPTVSIFPPSSEQLTSGGASVVCFLNNFTHCQELQQDECS
EbovGPscFv16-1   TKLEVKRADAAPTVSIFPPSSEQLTSGGASVVCFLNNFTHCQELQQDECS
EbovGPscFv3-2    TKLEIKRADAAPTVSIFPPSSEQLTSGGASVVCFLNNFTHCQELQQDECS
EbovGPscFv22-1   TKLEIKRADAAPTVSIFPPSSEQLTSGGASVVCFLNNFTHCQELQQDECS
                 ****::********************************************
```

S1 Fig. Alignment of the derived aminoacids sequences of the randomly chosen scFvs from linked antibody library and complementary determining regions (CDRs). FRs and CDRs are determined by the IMGT information system. Diversity was found predominantly in the CDR regions. A normal 20 amino acid linker [(G4S)4] joins the VH and VL chains. Alignments were **colour coded** according to residue property groups. AVFPMILW-red, DE-blue, RK-magenta, STYHCNGQ-green, others-grey.

Symbols in the alignment are as follows: (*) indicates where there is a conserved amino acid; (:) indicates an amino acid position with conserved similarity; (.) indicates a semi-conserved amino substitution has occurred; (-) indicates spaces introduced to optimise the alignment.

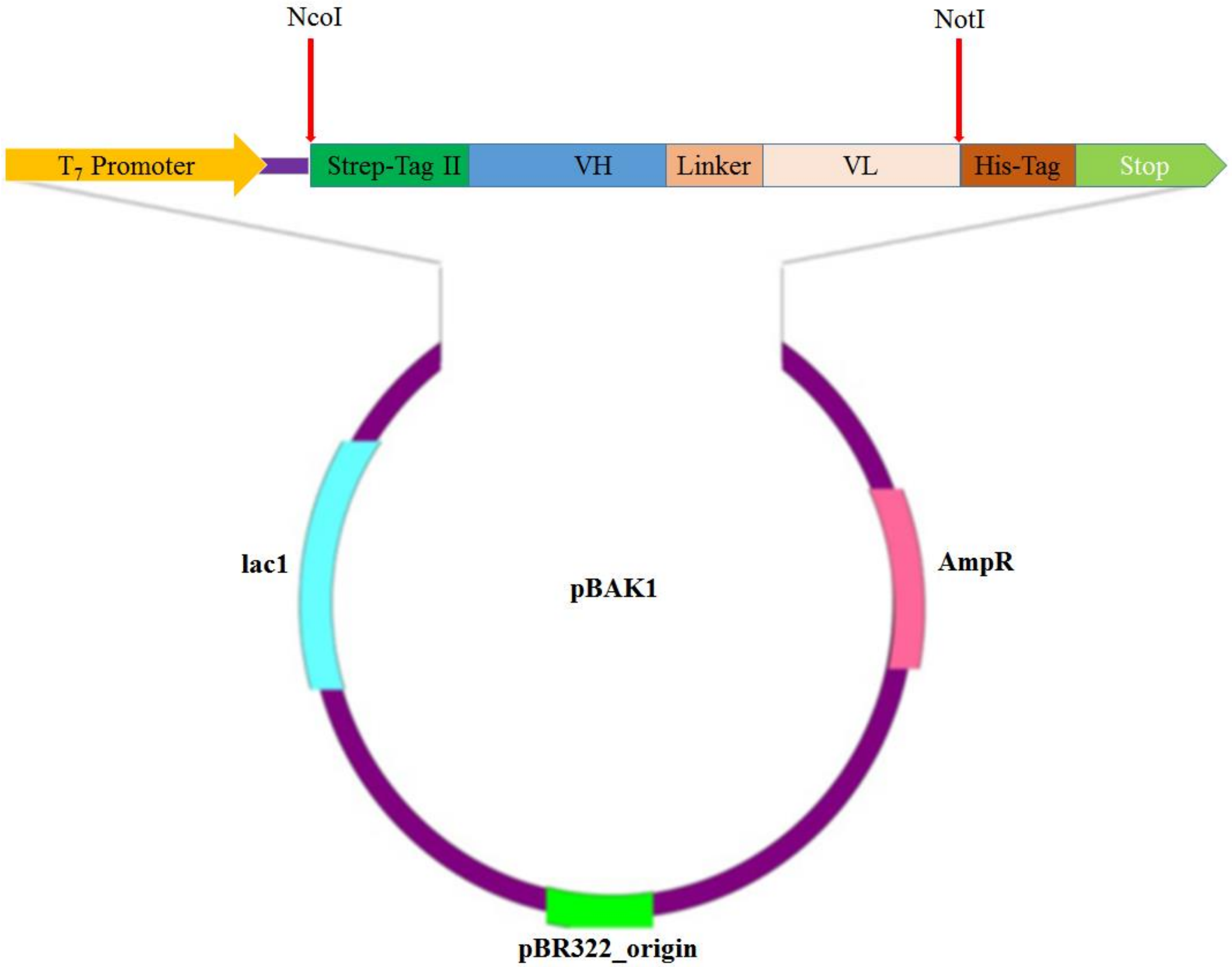

S2 Fig. Schematic diagram of the pBAK1 (*anti-EbovGP-his-ScFv*) expression vector. The position of the enzymatic cleavage is indicated by the *red arrow*. The gene encoding anti-EbovGP-his-ScFv protein was inserted into the pBAK1 vector under the control of the T7lac promoter, in frame with a strep tag II.

S1 Table

Nucleotide sequences of primers used.

**Primer name**	**Primer sequence (5' – 3')**
	**VH primers**
MVH_F1	CGAGAAGACCGGCAGCGGTGGGGCAGAGCTTGTGAAGCCA
MVH_F2	CGAGAAGACCGGCAGCGGTGGAGGAGGCTTGATGCAACCT
MVH_F3	CGAGAAGACCGGCAGCGGTGGACCTGAGCTGGAGATGCCT
MVH_F4	CGAGAAGACCGGCAGCGGTGGACCTGGCCTGGTGAGACCT
MVH_F5	CGAGAAGACCGGCAGCGGTGGGGGAGGCTTAGTGAAGCCT
MVH_F6	CGAGAAGACCGGCAGCGGTGGGGCAGAGCTTGTGAAGCCA
MVH_F7	CGAGAAGACCGGCAGCGGTGGAGGGGGCTTGGTACAGCCT
MVH_F8	CGAGAAGACCGGCAGCGGTGGGGCAGAGCTTGTGAGGTCA
MVH_R1	GGAGCCGCCGCCGCCGCCAGAACCACCACCACC***GGATCC***ACCACCACCCGAG GAAACGGTGACCGTGGT
MVH_R2	GGAGCCGCCGCCGCCGCCAGAACCACCACCACC***GGATCC***ACCACCACCCGAG GAGACTGTGAGAGTGGT
MVH_R3	GGAGCCGCCGCCGCCGCCAGAACCACCACCACC***GGATCC***ACCACCACCCGCA GAGACAGTGACCAGAGT
MVH_R4	GGAGCCGCCGCCGCCGCCAGAACCACCACCACC***GGATCC***ACCACCACCCGAG GAGACGGTGACTGAGGT
	**VL primers**
MVL_F1	GGCGGCGGCGGCTCCGGTGGTGGT***GGATCC***GCAATCATGTCTGCATCTCC
MVL_F2	GGCGGCGGCGGCTCCGGTGGTGGT***GGATCC***GCCTCCCTATCTGTATCTGTG
MVL_F3	GGCGGCGGCGGCTCCGGTGGTGGT***GGATCC***GCCTCCCTATCTGCATCTGTG
MVL_F4	GGCGGCGGCGGCTCCGGTGGTGGT***GGATCC***CTCACTTTGTCGGTTACCATT
MVL_F5	GGCGGCGGCGGCTCCGGTGGTGGT***GGATCC***TCAGCCTCTTTCTCCCTGGGA
MVL_F6	GGCGGCGGCGGCTCCGGTGGTGGT***GGATCC***TCCTCCCTGAGTGTGTCAGCA
MVL_F7	GGCGGCGGCGGCTCCGGTGGTGGT***GGATCC***CTCTCCCTGCCTGTCAGTCTT
MVL_F8	GGCGGCGGCGGCTCCGGTGGTGGT***GGATCC***CTCTCCCTGCCTGTCAGTCTT
MKR	AGAACACTCATTCCTGTTGAAGCTCTTGACAATGGGTGAAGTTG
MKR_NotI	AGT**GCGGCCGC**AGAACACTCATCCTGTTGAAGCTCTTGACAATGGGTGAAGT TG
	**Strep Tag II**
KzSTREPII	CGAATTCCACCATGGCC TGG AGC CAT CCG CAG TTC GAG AAG ACC GGC AGC GG
	**T7 promoter**
RDT7	CTATAGAAGG GTAATACGACTCACTATAGGGCGAATTCCACCATGGCC

S2 Table
Cross-reactivity profiles of anti-EBOV GP scFvs from ELISA result.

**scFv**	**GP_{EBOV}**	**GP_{SUDV}**	**GP_{RESTV}**	**GP_{BDBV}**	**GP_{TAFV}**	**GP_{MARV}**
3-2	+	+	-	-	+	+
4-2	+++	+++	++	++	++	+++
10-3	-	-	-	-	+	+
13-1	+	-	-	-	-	+
16-1	+	+	-	-	++	+
18-1	+	+	++	++	++	+
22-1	++	+++	++	++	++	+++
27-5	-	-	-	-	-	-
35-2	++	++	+	-	+	++
39-1	++	++	-	+	+	++
Mouse anti-Zaire ebola antibody 6D8	+++					
Mouse anti-Sudan ebola virus antibody		+++				
Rabbit anti-Reston GP polyclonal antibody			+++			
Rabbit anti-Bundibugyo GP polyclonal antibody				+++		
Rabbit anti-MARV GP polyclonal antibody						+++
Rabbit anti-Tai Forest virus GP IgG					+++	
XfPilB21	-	-	-	-	-	-

†: GPs of each virus species were used as antigens. *: scFv reactivity was evaluated based on ELISA OD450. +++: OD>1.0; ++: OD ≥ 1.0; +: 0.3 < OD < 1.0; -: OD ≤ 0.3. XfPilBscFv21: Negative control. Mouse anti-Zaire ebola antibody 6D8, Mouse anti-Sudan ebola virus antibody, Rabbit anti-Reston GP polyclonal antibody, Rabbit anti-Bundibugyo GP polyclonal antibody, Rabbit anti-MARV GP polyclonal antibody, and Rabbit anti-Tai Forest virus GP IgG: Positive controls.

S3 Table 3

% CV & Bias around calculated EC-50. Accuracy and precision around the calculated EC-50 values are within acceptable level.

scFv4-2 vs GP_{EBOV}				scFv4-2 vs GP_{SUDV}			scFv4-2 vs GP_{MARV}		
**µg/mL**	**Mean Absorbance**	**%CV of calculated conc**	**%BIAS**	**Mean Absorbance**	**%CV of calculated conc**	**%BIAS**	**Mean Absorbance**	**%CV of calculated conc**	**%BIAS**
30	2.748	21.0	1.2	2.121	5.8	-5.1	2.847	31.8	-1.9
20	2.709	34.3	10.4	2.030	9.0	4.7	2.816	4.1	2.4
15	2.667	3.9	0.1	1.916	1.7	5.1	2.756	13.1	5.9
10	2.523	11.5	4.4	1.613	2.4	-5.4	2.523	8.4	-0.4
5	1.735	3.3	-0.4	1.117	4.9	2.2	1.627	7.2	0.3
1	0.143	1.6	8.5	0.170	2.0	-3.7	0.067	1.8	-0.4
0.1	0.049	97.9	3.8	0.006	7.1	-0.2	0.0	6.3	18.1
0.01	0.034	Range?	Range?	0.005	12.7	776.8	0.0	6.0	1019.1

S4 Table. % CV & Bias around calculated EC-50. Accuracy and precision around the calculated EC-50 values are within acceptable level.

scFv4-2 vs GP_{BDBV}				scFv4-2 vs GP_{RESTV}			scFv4-2 vs GP_{TAFV}		
**µg/ml**	**Mean Absorbance**	**%CV of calculated conc**	**%BIAS**	**Mean Absorbance**	**%CV of calculated conc**	**%BIAS**	**Mean Absorbance**	**%CV of calculated conc**	**%BIAS**
30	1.286	4.2	-0.5	1.349	10.1	1.0	2.065	6.4	-2.6
20	1.199	13.0	1.2	1.266	15.0	1.3	1.918	16.4	4.8
15	1.087	5.1	0.3	1.167	0.6	0.6	1.765	5.7	3.9
10	0.832	3.4	0.1	0.930	5.5	0.1	1.426	2.7	-6.2
5	0.328	0.3	-0.05	0.424	0.2	-0.1	0.984	0.8	3.7
1	0.020	4.9	5.4	0.033	5.5	9.8	0.179	1.5	-5.9
0.1	0.009	54.4	10.9	0.012	17.5	12.0	0.011	4.6	14.7
0.01	0.008	Range?	Range?	0.008	0	5755.6	0.002	7.0	402.7

S5 Table

% CV & Bias around calculated EC-50. Accuracy and precision around the calculated EC-50 values are within acceptable level.

	scFv22-1 vs GP_{EBOV}				scFv4-2 vs GP_{SUDV}				scFv4-2 vs GP_{MARV}		
µg/ml	Mean Absorbance	%CV of calculated conc	%BIAS		Mean Absorbance	%CV of calculated conc	%BIAS		Mean Absorbance	%CV of calculated conc	%BIAS
30	2.711	6.3	0.2		1.937	23.9	3.2		1.636	12.8	0.8
20	2.497	13.2	4.0		1.763	4.3	0.3		1.592	8.2	2.3
15	2.261	6.2	0.263		1.593	5.6	0.3		1.507	9.0	0.2
10	1.835	5.3	-5.0		1.301	0.9	0.368		1.269	6.5	0.5
5	1.235	5.4	5.5		0.749	1.7	-0.697		0.530	9.0	-0.1
1	0.184	0.8	-18.0		0.124	3.6	5.3		0.020	4.2	18.2
0.1	0.035	12.0	16.0		0.020	23.4	-16.0		0.006	16.2	18.6
0.01	0.059	73.8	3133.1		0.012	0	307.5		0.001	Range?	Range?

S6 Table.

% CV & Bias around calculated EC-50. Accuracy and precision around the calculated EC-50 values are within acceptable level.

	scFv4-2 vs GP_{BDBV}				scFv4-2 vs GP_{RESTV}				scFv4-2 vs GP_{TAFV}		
µg/ml	Mean Absorbance	%CV of calculated conc	%BIAS		Mean Absorbance	%CV of calculated conc	%BIAS		Mean Absorbance	%CV of calculated conc	%BIAS
30	1.838	9.1	-5.1		1.872	8.2	-1.4		1.508	11.2	-4.6
20	1.688	3.0	0.3		1.706	2.1	1.2		1.366	3.1	7.9
15	1.465	1.0	3.6		1.513	12.3	1.9		1.141	1.9	1.0
10	0.813	0.4	-3.0		1.134	0.8	-1.0		0.800	1.7	-6.4
5	0.231	1.5	10.4		0.521	1.0	0.3		0.445	14.3	5.0
1	0.027	9.4	2.7		0.060	9.6	18.7		0.069	11.8	8.9
0.1	0.017	Range?	Range?		0.019	1.8	4.3		0.023	85.0	-24.9
0.01	0.004	Range?	Range?		0.008	Range?	Range?		0.012	Range?	Range?

S7 Table

Apparent affinity and maximal binding of EBOVGP scFvs to GP_{ZEBOV}, GP_{SUDV}, GP_{RESTV}, GP_{BDBV}, GP_{TAFV} and GP_{MARV}.

**GP**	**scFv4-2**		**scFv22-1**	
	**K_d.app (µg)**	**B_{max} signal (10^5)**	**K_d.app (µg)**	**B_{max} signal (10^5)**
ZEBOV	4.8 ± 1.5	3.5 ± 0.3	9.3 ± 1.2	3.7 ± 0.2
SUDV	6.6 ± 0.9	2.8 ± 0.1	10.8 ± 1.6	2.8 ± 0.2
RESTV	12.2 ± 3.4	2.1 ± 0.2	17.4 ± 4.2	3.2 ± 0.4
BDBV	15.1 ± 4.8	2.2 ± 0.3	38 ± 23	4.6 ± 1.8
TAFV	8.2 ± 0.8	2.8 ± 0.1	19.3 ± 4.3	2.7 ± 0.3
MARV	5.9 ± 1.8	3.8 ± 0.3	10.8 ± 3.8	2.5 ± 0.3

The values for apparent affinity (K_d,*app*) and maximal binding (B_{max}, shown as relative absorbance units) were derived from the ELISAs with GP_{ZEBOV}, GP_{SUDV}, GP_{RESTV}, GP_{BDBV}, GP_{TAFV} and GP_{MARV}. Values are averages (and ranges) of three experiments.
